# Supplementary material for: Transcriptome Analysis of Cisplatin, Cannabidiol, and Intermittent Serum Starvation Alone and in Various Combinations on Colorectal Cancer Cells
Source: Int J Mol Sci. 2023 Sep 29;24(19):14743. doi: 10.3390/ijms241914743 (PMC10572413; doi:10.3390/ijms241914743)
Supplement: Supplementary file 1 [file ijms-24-14743-s001.zip › ijms-2642649-supplementary.pdf]

Table S1. Calculated CI and treatment interactions for various ISS, CBD, and cisplatin combinations in the HCT-116 CRC cell line. Data was generated using CompuSyn software.

Intermittent Serum Starvation	CBD	Cisplatin	Fa	CI	Interaction effect
-	IC95	IC95	0.99	1.2	Slight antagonism
-	IC75	IC75	0.29	12.1	*Strong antagonism
IC50	-	IC70	0.61	1.3	Moderate antagonism
IC50	-	IC50	0.63	0.9	*Nearly additive
IC50	IC70	-	0.99	0.03	Very strong synergism
IC50	IC50	-	0.86	0.24	*Strong synergism
IC50	IC70	IC50	0.97	0.097	Very strong synergism
IC50	IC50	IC50	0.94	0.17	*Strong synergism

* Treatment dose combinations selected for mRNA sequencing analysis
